# Hexa-μ_2_-acetato-hexa-*n*-butyl­hexa-μ_3_-oxido-tin(IV) toluene monosolvate

**DOI:** 10.1107/S160053681204888X

**Published:** 2012-12-05

**Authors:** Martin Reichelt, Hans Reuter

**Affiliations:** a Institut für Chemie neuer Materialien, Anorganische Chemie II, Universität Osnabrück, Barbarastrasse 7, 49069 Osnabrück, Germany

## Abstract

The title compound, [Sn_6_(C_4_H_9_)_6_(CH_3_COO)_6_O_6_]·C_7_H_8_, has one half-toluene mol­ecule and one half-organotin mol­ecule in the asymmetric unit. The latter is situated about an inversion centre and belongs to the class of hexa­meric monoorganooxo­tin carboxyl­ates with a hexa­gonal prismatic or ‘drum-like’ motif of the central tin–oxygen core. Two Sn_3_O_3_ rings in a flat-chair conformation are linked *via* six Sn—O bonds and six bridging acetate groups. All Sn atoms have approximate octa­hedral coordination geometry. The Sn—O bonds which are *trans* to the alkyl group are significantly shorter than the others. One butyl group is disordered over two different sites, with occupancies of 0.9:0.1. Very large atomic displacement parameters of the toluene mol­ecule indicate an unresolvable disorder about the twofold axis.

## Related literature

For an overview of the synthesis of organotin carboxyl­ates, see: Mehrotra & Bohra (1983 ▶). For an overview on compositions and structure types of organotin carboxyl­ates, see: Tiekink (1991 ▶). For structural details on hexa­meric, ‘drum-like’ monoorganooxotin acetates, see: Day *et al.* (1988 ▶); Kuan *et al.* (2002 ▶); Beckmann *et al.* (2004 ▶). For ‘ladder-type’ monoorganooxotin carboxyl­ates, see: Day *et al.* (1988 ▶). For the static *trans* strengthening in alkyltin(IV) halides, see: Buslaev *et al.* (1989 ▶); Reuter & Puff (1992 ▶).
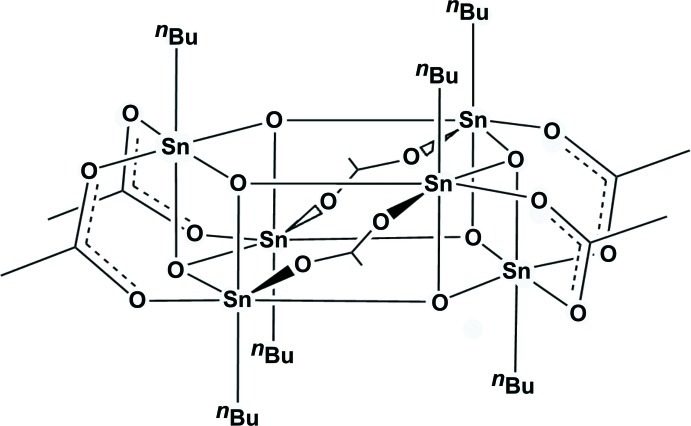


## Experimental

### 

#### Crystal data

[Sn_6_(C_4_H_9_)_6_(C_2_H_3_O_2_)_6_O_6_]·C_7_H_8_
*M* *_r_* = 1597.21
Monoclinic, 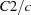

*a* = 23.4154 (8) Å
*b* = 15.5832 (6) Å
*c* = 16.1012 (6) Å
β = 93.926 (2)°
*V* = 5861.3 (4) Å^3^
*Z* = 4
Mo *K*α radiation
μ = 2.58 mm^−1^
*T* = 150 K
0.30 × 0.22 × 0.10 mm

#### Data collection

Bruker APEXII CCD diffractometer
Absorption correction: multi-scan (*SADABS*; Bruker, 2009 ▶) *T*_min_ = 0.509, *T*_max_ = 0.783
76854 measured reflections
6788 independent reflections
5890 reflections with *I* > 2σ(*I*)
*R*_int_ = 0.035

#### Refinement

*R*[*F* ^2^ > 2σ(*F* ^2^)] = 0.020
*wR*(*F* ^2^) = 0.046
*S* = 1.05
6788 reflections
325 parameters
6 restraints
H-atom parameters constrained
Δρ_max_ = 0.65 e Å^−3^
Δρ_min_ = −0.61 e Å^−3^

### 

Data collection: *APEX2* (Bruker, 2009 ▶); cell refinement: *SAINT* (Bruker, 2009 ▶); data reduction: *SAINT*; program(s) used to solve structure: *SHELXS97* (Sheldrick, 2008 ▶); program(s) used to refine structure: *SHELXL97* (Sheldrick, 2008 ▶); molecular graphics: *DIAMOND* (Brandenburg, 2006 ▶); software used to prepare material for publication: *SHELXTL* (Sheldrick, 2008 ▶).

## Supplementary Material

Crystal structure: contains datablock(s) I, global. DOI: 10.1107/S160053681204888X/fj2609sup1.cif

Structure factors: contains datablock(s) I. DOI: 10.1107/S160053681204888X/fj2609Isup2.hkl

Additional supplementary materials:  crystallographic information; 3D view; checkCIF report

## Figures and Tables

**Table 1:** Selected torsion angles (°)

Sn2—O2—Sn3—O1^i^	−25.88 (11)
O2—Sn3—O1^i^—Sn1^i^	24.87 (11)
Sn3—O1^i^—Sn1^i^—O3^i^	−24.64 (11)
O1^i^—Sn1^i^—O3^i^—Sn2	24.53 (11)
Sn1^i^—O3^i^—Sn2—O2	−24.89 (11)
O3^i^—Sn2—O2—Sn3	25.96 (11)

Symmetry code: (i) 
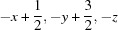
.

**Fig. 1.**
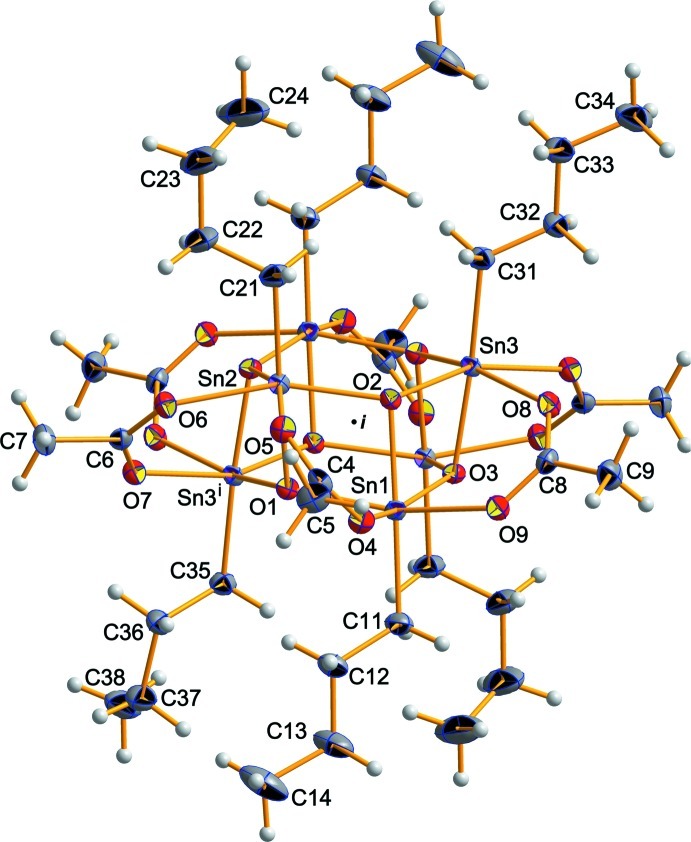
Ball-and-stick model of title compound with the atomic numbering scheme used; with exception of the hydrogen atoms, which are shown as spheres with common isotropic radius, all other atoms are represented as thermal displacement ellipsoids showing the 50% probability level of the corresponding atom; the statistically disordered n-butyl group of Sn3 is shown in case the major (90%) one at Sn3 and in case of the minor one (10%) at Sn3i; symmetry code: (i) -x + 1/2, -y + 3/2, -z.

**Fig. 2.**
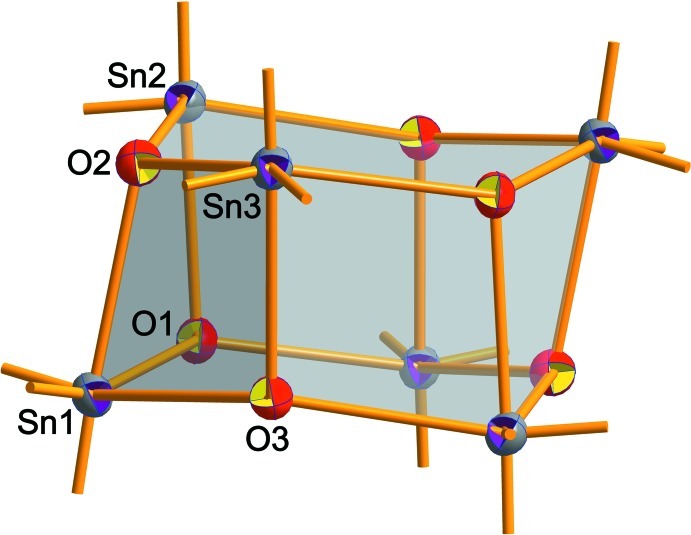
Ball-and-stick model of the central, drum-like tin-oxygen core of the title compound showing the flat chair-like conformation of the six-membered tin-oxygen rings of the as well as the non-planarity of the tin-oxygen trapezoids; with exception of the hydrogen atoms, which are shown as spheres with common isotropic radius, all other atoms are represented as thermal displacement ellipsoids showing the 50% probability level of the corresponding atom; in cases of the octahedrally coordinated the additional bonds are drawn as shortened sticks to visualize their orientation.

**Fig. 3.**
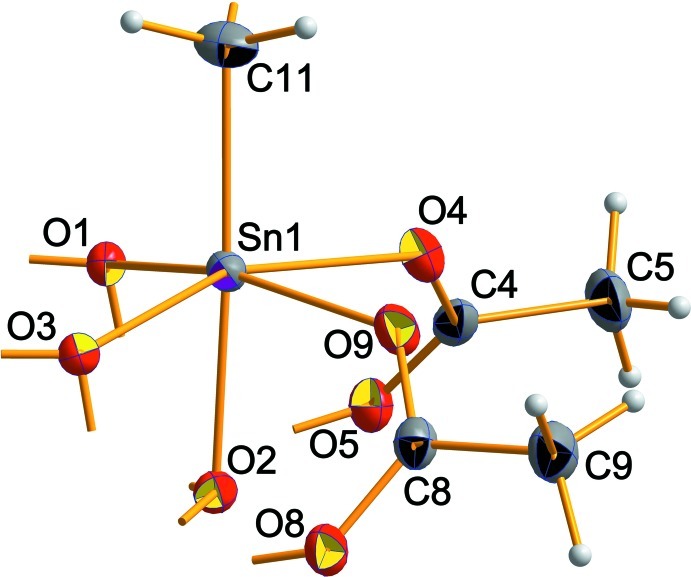
Ball-and-stick model of the octahedral coordination sphere of Sn1; with exception of the hydrogen atoms, which are shown as spheres with common isotropic radius, all other atoms are represented as thermal displacement ellipsoids showing the 50% probability level of the corresponding atom; in cases (nBu, µ3-O, O2CCH3) that atoms form additional bonds these are drawn as shortened sticks to show the orientation.

**Fig. 4.**
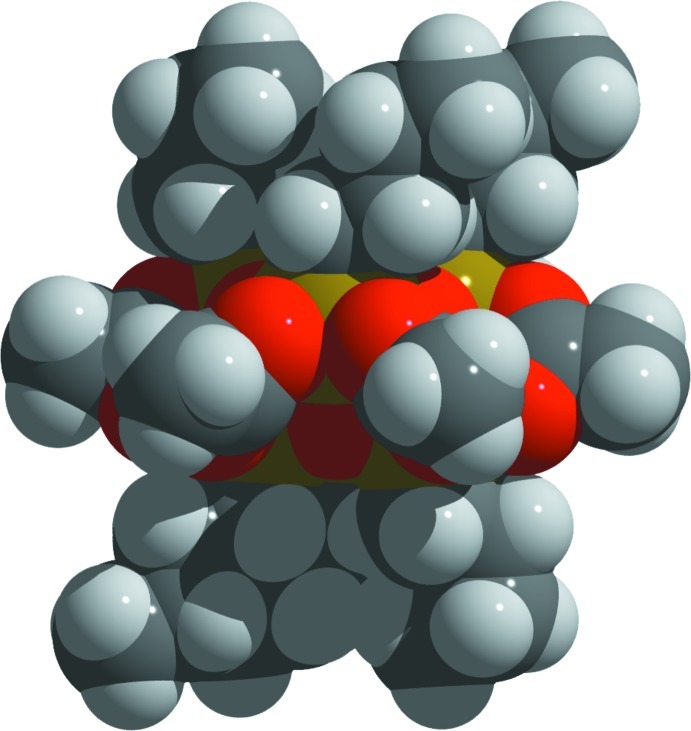
Space-filling model of the title compound showing the cylindrical shape of the molecule with the belt of acetate groups surrounding the central tin-oxygen framework and its coverage through the n-butyl groups.

**Fig. 5.**
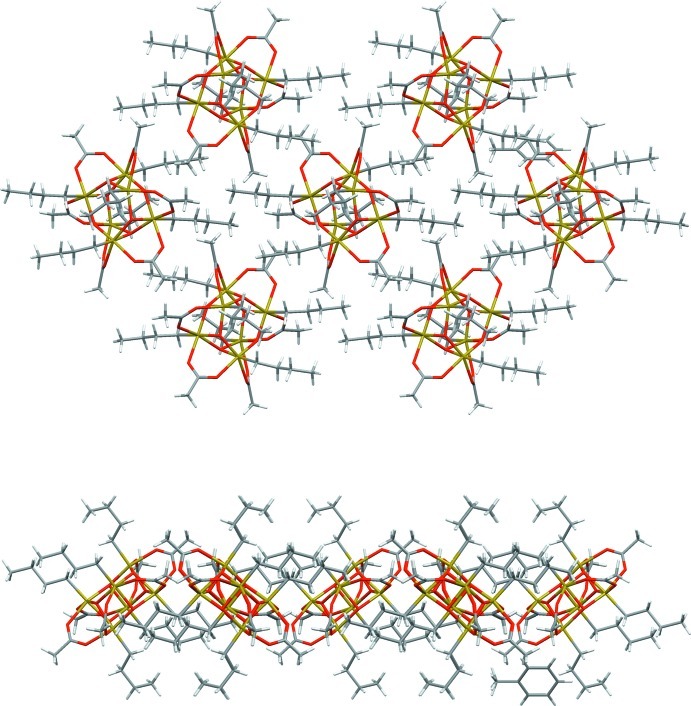
Detail of the three-dimensional arrangement of the hexameric molecules, showing the sheets (top view above, side view below) formed by van-der Waals interaction.

**Fig. 6.**
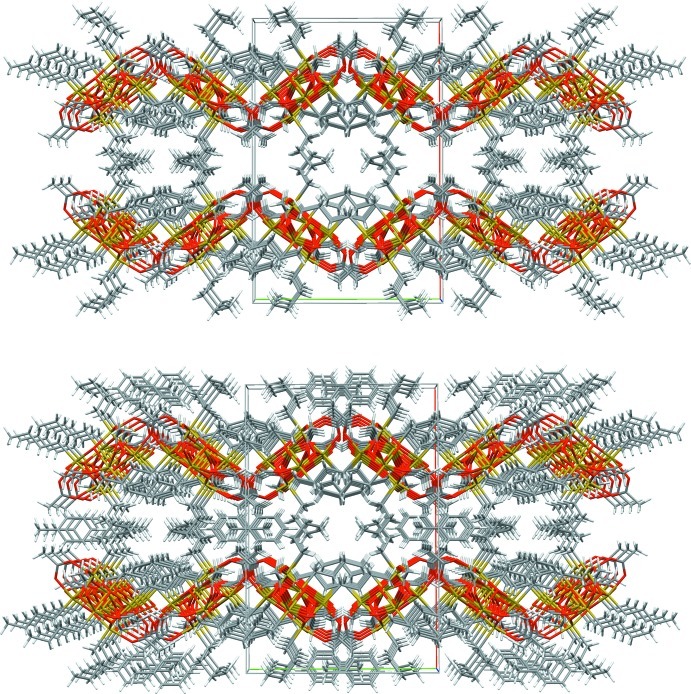
Detail of the three-dimensional arrangement of the hexameric molecules, showing the stacking of the sheets and the open space between them (above, a) and its filling by the toluene molecules (below, b).

## supplementary crystallographic information

### Comment

The synthesis of organotin(IV) carboxylates is well established since a long time (Mehrotra & Bohra, 1983) and many of their crystal structures have been investigated into detail (Tiekink, 1991). Thus, in the case of triorganotin moieties *R_3_*Sn the corresponding carboxylates are of general formula *R*_3_Sn(O_2_C*R*') with (O_2_C*R*') representing the carboxylate ion. Diorganotin oxides, *R*_2_SnO, most often insoluble in indifferent organic solvents, can be easily dissolved in a large number of carboxylic acids, *R*'COOH, giving rise to the formation of carboxylates with a lot of different compositions and a tremendous diversity of molecular structures. Applying the same conditions to monoorganotin sesquioxides, *R*SnO_1,5_, also insoluble in non-complexing organic solvents results in the formation of monoorganooxotin carboxylates with "ladder"-type structures of composition (RSn)_6_O_4_(O_2_CR')_10_ (Day *et al.*,1988) or, more frequently, to hexanuclear compounds [*R*SnO(O_2_C*R*')]_6_. In the latter case, the structure is dominated by a hexagonal prismatic or "*drum*"-like arrangement of the tin-oxygen core as was demonstrated in the case of acetates (*R*' = CH_3_) for *R* = *^i^*Pr (Kuan *et al.*, 2002), *R* = tmsm (Beckmann *et al.*, 2004), and *R* = Me (Day *et al.*,1988). By dissolving n-butylstannonic acid of idealized formula *^n^*BuSnO(OH) in a mixture of toluene/acetic acid and removing the resulting water by use of a Dean-Stark apparatus we were able to obtain single crystals of the corresponding *R* = *n-*butyl hexamer as the 1:1 toluene solvate, [*^n^*BuSnO(OAc)]_6_*x* C_7_H_8_.

The asymmetric unit of the title compound consists of a centrosymmetric hexamer (Fig. 1) with i at (1/4,3/4,0) and a toluene molecule of site symmetry 2. As usual for the constitution (Fig. 2) of the *drum*, the six-membered tin-oxygen rings forming the bases are not planar but adopt a flat chair-conformation with the torsion angles listed in Tab. 1. Both rings are rotated through 60° against each other so that each oxygen atom bonds to a tin atom of the adjacent ring. The six sides of the *drum* consist of tin oxygen trapezoids with small angles at tin [77.88 (6)° - 78.34 (6)°, mean value 78.0 (2)°] and larger ones at oxygen [99.79 (6)° - 100.55 (6)°, mean value 100.1 (3)°]. These four-membered rings are also non-planar but bent [18.19 (5)°-18.99 (4)°] along the O···O diagonals that are 2.625 (2) - 2.636 (2) Å long in contrast to the Sn··· Sn diagonals that are much more longer [3.1980 (2) - 3.2041 (2) Å]. Both tin atoms of these six four-membered tin oxygen rings are bridged by acetate groups with carbon oxygen distances that are almost equal [1.256 (3) - 1.267 (3) Å, mean value 1.263 (5) Å] indicating their symmetrical coordination mode (Fig. 3).

In summary, all tin atoms are octahedrally coordinated by the *n*-butyl ligand, three oxygen atoms of the *drum* and two oxygen atoms of two different acetate groups (Fig. 2). The distortions of the octahedra can be described by carbon-tin-oxygen axes that are slightly bent [175.48 (7)° - 177.18 (8)°], and bond angles of the organic groups to their *cis*-oriented neighboring oxygen atoms that are considerably widened [91.90 (7)° - 100.53 (8)°].

While the tin-carbon bonds are very similar [2.125 (2) to 2.129 (2) Å, mean value: 2.128 (2) Å] tin-oxygen bond lengths fall into two clearly different groups. The longer ones [2.162 (2) - 2.172 (2) Å, mean value: 2.167 (5) Å] are those to the bridging acetate groups. The narrow range is in correspondence with the narrow range of carbon oxygen bonds, described above, indicating a symmetrical bonding of the acetate groups. The shorter ones [2.080 (2) - 2.097 (2) Å] arise from the µ_3_-oxygen atoms within the *drum*. They can be subdivided into those that are *trans* to the organic ligand and significantly shorter [2.080 (2) - 2.085 (2) Å, mean value: 2.083 (3) Å] and those that are somewhat longer arising from the corresponding *cis*-oriented oxygen atoms [2.0882 (2) - 2.0969 (2) Å, mean value: 2.092 (4) Å]. This strengthening although less marked is similar to the static *trans*-strengthening observed in the case of alkyl tin halides (Buslaev *et al.*, 1989), Reuter & Puff, 1992).

Intermolecular interactions of the cylindrical hexamers are limited to van-der-Waals ones because the accessibility of the polar tin-oxygen core is restricted (Fig. 4). As a result of these weak interactions, molecules are arranged in planar hexagonal nets (Fig. 5a) with two different orientations of the drum (Fig. 5b). Between these nets large apolar channels with large cavities exist (Fig. 6a) which partially are fulfilled by the solvent molecules (Fig. 6b).

### Experimental

1.24 g (6 mmol) n-butylstannonic acid (Gelest, Inc.) and 0.36 g (6 mmol) glacial acetic acid (Fluka) were dissolved in 120 ml toluene. The mixture was heated under reflux for 6 h. The water formed in the reaction was removed by a Dean-Stark apparatus. The reaction mixture was filtered and the solvent evaporated overnight at room temperature. After evaporation colourless block shaped crystals was obtained.

^1^H-NMR (Bruker AVANCE DPX, 250 MHz, CDCl_3_) δ [p.p.m.]: 0.91 (t, 3H, CH_3_); 1.57–1.75 (m, 2H); 1.18–1.55 (m, 4H); 2.09 (s, 3H, CH_3_COO).

{^1^H}-^13^C-NMR (Bruker AVANCE DPX, 250 MHz, CDCl_3_) δ [p.p.m.], *^n^*J(^13^C-^119/117^Sn) [Hz]: 26.74 (α-CH_2_, *n* = 1, 1191.9/1139.0); 27.02 (β-CH_2_, *n* = 2, 57.4); 26.48 (γ-CH_2_, *n* = 3, 195.9/187.6); 13.48 (CH_3_, *n* = 4, 14.8); 24.22 (CH_3_—COO); 179.64 (CH_3_—COO, *n* = 2, 30.3).

A suitable single-crystal was selected under a polarization microsope and mounted on a 50 µm MicroMesh MiTeGen Micromount^TM^ using FROMBLIN Y perfluoropolyether (LVAC 16/6, Aldrich).

### Refinement

The *n*-butyl group at Sn3 is statistically disordered resulting in two different conformations with occupancies 0.9/0.1. In order to get a reliable structure model carbon-carbon bonds of the minor part were restraint to a common refined value of 1.516(*x*) Å and their anisotropic displacement parameters fit to those of the corresponding carbon atoms of the major part. Although the hydrogen atoms of the non-disordered carbon atoms could clearly identified in difference Fourier synthesis, all were idealized and refined at calculated positions riding on the carbon atoms with C—H distances of 0.98 Å (–CH_3_), 0.98 (–CH_2_–) and 0.95 Å (H_aromatic_). Carbon atoms of the solvent molecule show high anisotropic displacement parameters as a result of high thermal motion or more probably as a result of its statistic disorder about the twofold axis giving rise to unusual bond lengths and angles.

### Crystal data

**Table:**

[Sn_6_(C_4_H_9_)_6_(C_2_H_3_O_2_)_6_O_6_]·C_7_H_8_	*F*(000) = 3128
*M**_r_* = 1597.21	*D*_x_ = 1.810 Mg m^−^^3^
Monoclinic, *C*2/*c*	Mo *K*α radiation, λ = 0.71073 Å
Hall symbol: -C 2yc	Cell parameters from 9091 reflections
*a* = 23.4154 (8) Å	θ = 2.6–30.0°
*b* = 15.5832 (6) Å	µ = 2.58 mm^−^^1^
*c* = 16.1012 (6) Å	*T* = 150 K
β = 93.926 (2)°	Block, colourless
*V* = 5861.3 (4) Å^3^	0.30 × 0.22 × 0.10 mm
*Z* = 4	

### Data collection

**Table:**

Bruker APEXII CCD diffractometer	6788 independent reflections
Radiation source: fine-focus sealed tube	5890 reflections with *I* > 2σ(*I*)
Graphite monochromator	*R*_int_ = 0.035
φ and ω scans	θ_max_ = 28.0°, θ_min_ = 2.5°
Absorption correction: multi-scan (*SADABS*; Bruker, 2009)	*h* = −30→28
*T*_min_ = 0.509, *T*_max_ = 0.783	*k* = −20→19
76854 measured reflections	*l* = −20→21

### Refinement

**Table:**

Refinement on *F*^2^	Primary atom site location: structure-invariant direct methods
Least-squares matrix: full	Secondary atom site location: difference Fourier map
*R*[*F*^2^ > 2σ(*F*^2^)] = 0.020	Hydrogen site location: inferred from neighbouring sites
*wR*(*F*^2^) = 0.046	H-atom parameters constrained
*S* = 1.05	*w* = 1/[σ^2^(*F*_o_^2^) + (0.0188*P*)^2^ + 9.9195*P*] where *P* = (*F*_o_^2^ + 2*F*_c_^2^)/3
6788 reflections	(Δ/σ)_max_ = 0.002
325 parameters	Δρ_max_ = 0.65 e Å^−^^3^
6 restraints	Δρ_min_ = −0.61 e Å^−^^3^

### Special details

**Table:**

Geometry. All e.s.d.'s (except the e.s.d. in the dihedral angle between two l.s. planes) are estimated using the full covariance matrix. The cell e.s.d.'s are taken into account individually in the estimation of e.s.d.'s in distances, angles and torsion angles; correlations between e.s.d.'s in cell parameters are only used when they are defined by crystal symmetry. An approximate (isotropic) treatment of cell e.s.d.'s is used for estimating e.s.d.'s involving l.s. planes.
Refinement. Refinement of *F*^2^ against ALL reflections. The weighted *R*-factor *wR* and goodness of fit *S* are based on *F*^2^, conventional *R*-factors. *R* are based on *F*, with *F* set to zero for negative *F*^2^. The threshold expression of *F*^2^ > σ(*F*^2^) is used only for calculating *R*-factors(gt) *etc*. and is not relevant to the choice of reflections for refinement. *R*-factors based on *F*^2^ are statistically about twice as large as those based on *F*, and *R*- factors based on ALL data will be even larger.

### Fractional atomic coordinates and isotropic or equivalent isotropic displacement parameters (Å^2^)

**Table:**

	*x*	*y*	*z*	*U*_iso_*/*U*_eq_	Occ. (<1)
Sn1	0.218624 (6)	0.777418 (9)	0.142503 (8)	0.01627 (4)	
Sn2	0.343645 (6)	0.788062 (10)	0.073955 (8)	0.01612 (4)	
Sn3	0.238831 (6)	0.599363 (9)	0.049412 (8)	0.01573 (4)	
O1	0.26833 (6)	0.86006 (10)	0.07422 (8)	0.0175 (3)	
O2	0.28513 (6)	0.69761 (10)	0.11156 (8)	0.0172 (3)	
O3	0.17758 (6)	0.69645 (10)	0.05325 (9)	0.0171 (3)	
O4	0.27161 (7)	0.81598 (10)	0.25130 (9)	0.0228 (4)	
O5	0.35980 (7)	0.81837 (10)	0.20439 (9)	0.0223 (4)	
C4	0.32520 (11)	0.82347 (14)	0.26131 (13)	0.0208 (5)	
C5	0.35026 (11)	0.84157 (18)	0.34786 (14)	0.0309 (6)	
H5A	0.3916	0.8310	0.3507	0.064 (3)*	
H5B	0.3324	0.8040	0.3874	0.064 (3)*	
H5C	0.3432	0.9016	0.3620	0.064 (3)*	
O6	0.39045 (7)	0.90787 (10)	0.06712 (9)	0.0212 (3)	
O7	0.33111 (7)	0.98846 (10)	−0.01633 (9)	0.0204 (3)	
C6	0.37742 (10)	0.97521 (15)	0.02701 (13)	0.0194 (5)	
C7	0.42114 (11)	1.04577 (16)	0.03037 (15)	0.0276 (5)	
H7A	0.4479	1.0365	−0.0130	0.064 (3)*	
H7B	0.4422	1.0458	0.0851	0.064 (3)*	
H7C	0.4019	1.1011	0.0212	0.064 (3)*	
O8	0.21456 (7)	0.55655 (10)	0.17029 (9)	0.0223 (3)	
O9	0.19702 (7)	0.68149 (10)	0.23257 (9)	0.0229 (4)	
C8	0.20029 (10)	0.60108 (15)	0.23140 (13)	0.0202 (5)	
C9	0.18485 (12)	0.55366 (17)	0.30800 (14)	0.0303 (6)	
H9A	0.1954	0.5884	0.3574	0.064 (3)*	
H9B	0.2055	0.4990	0.3118	0.064 (3)*	
H9C	0.1435	0.5427	0.3047	0.064 (3)*	
C11	0.14968 (11)	0.86040 (16)	0.16782 (16)	0.0282 (6)	
H11A	0.1221	0.8613	0.1184	0.053 (3)*	
H11B	0.1297	0.8360	0.2146	0.053 (3)*	
C12	0.16665 (11)	0.95254 (16)	0.18958 (15)	0.0273 (5)	
H12A	0.1903	0.9527	0.2429	0.053 (3)*	
H12B	0.1905	0.9751	0.1461	0.053 (3)*	
C13	0.11608 (13)	1.01168 (18)	0.1972 (2)	0.0445 (8)	
H13A	0.0944	0.9926	0.2445	0.053 (3)*	
H13B	0.0903	1.0072	0.1459	0.053 (3)*	
C14	0.13350 (16)	1.10523 (19)	0.2104 (2)	0.0563 (10)	
H14A	0.1584	1.1103	0.2616	0.053 (3)*	
H14B	0.0992	1.1404	0.2151	0.053 (3)*	
H14C	0.1541	1.1250	0.1630	0.053 (3)*	
C21	0.41783 (11)	0.70950 (17)	0.07025 (15)	0.0288 (6)	
H21A	0.4323	0.6962	0.1281	0.110 (5)*	
H21B	0.4067	0.6546	0.0427	0.110 (5)*	
C22	0.46616 (13)	0.7496 (2)	0.0248 (2)	0.0513 (9)	
H22A	0.4788	0.8029	0.0540	0.110 (5)*	
H22B	0.4513	0.7655	−0.0322	0.110 (5)*	
C23	0.51778 (14)	0.6905 (2)	0.0190 (3)	0.0629 (10)	
H23A	0.5495	0.7237	−0.0030	0.110 (5)*	
H23B	0.5306	0.6707	0.0756	0.110 (5)*	
C24	0.50580 (17)	0.6140 (3)	−0.0354 (3)	0.0911 (16)	
H24A	0.4739	0.5814	−0.0149	0.110 (5)*	
H24B	0.5399	0.5775	−0.0343	0.110 (5)*	
H24C	0.4958	0.6329	−0.0926	0.110 (5)*	
C31	0.30603 (10)	0.50768 (13)	0.04935 (14)	0.0229 (5)	0.90
H31A	0.3264	0.5160	−0.0019	0.050 (3)*	0.90
H31B	0.3336	0.5200	0.0973	0.050 (3)*	0.90
C32	0.28870 (13)	0.41416 (12)	0.05386 (19)	0.0290 (7)	0.90
H32A	0.2640	0.3994	0.0035	0.050 (3)*	0.90
H32B	0.2660	0.4056	0.1029	0.050 (3)*	0.90
C33	0.33987 (14)	0.35436 (16)	0.0603 (2)	0.0412 (9)	0.90
H33A	0.3609	0.3601	0.0093	0.050 (3)*	0.90
H33B	0.3660	0.3722	0.1081	0.050 (3)*	0.90
C34	0.32403 (17)	0.26092 (18)	0.0712 (2)	0.0549 (10)	0.90
H34A	0.3020	0.2548	0.1205	0.050 (3)*	0.90
H34B	0.3590	0.2263	0.0783	0.050 (3)*	0.90
H34C	0.3009	0.2412	0.0218	0.050 (3)*	0.90
C35	0.30603 (10)	0.50768 (13)	0.04935 (14)	0.0229 (5)	0.10
H35A	0.3353	0.5282	0.0125	0.050 (3)*	0.10
H35B	0.3244	0.5028	0.1064	0.050 (3)*	0.10
C36	0.2853 (11)	0.4198 (7)	0.0203 (18)	0.0290 (7)	0.10
H36A	0.2532	0.4033	0.0540	0.050 (3)*	0.10
H36B	0.2695	0.4254	−0.0381	0.050 (3)*	0.10
C37	0.3279 (15)	0.346 (2)	0.0239 (18)	0.0412 (9)	0.10
H37A	0.3591	0.3610	−0.0122	0.050 (3)*	0.10
H37B	0.3084	0.2947	0.0002	0.050 (3)*	0.10
C38	0.3546 (15)	0.324 (2)	0.1096 (16)	0.0549 (10)	0.10
H38A	0.3673	0.3767	0.1386	0.050 (3)*	0.10
H38B	0.3875	0.2862	0.1040	0.050 (3)*	0.10
H38C	0.3263	0.2948	0.1416	0.050 (3)*	0.10
C41	0.5000	−0.0820 (4)	0.2500	0.100 (2)	
H41A	0.4606	−0.1030	0.2518	0.150*	0.50
H41B	0.5231	−0.1030	0.2989	0.150*	0.50
H41C	0.5163	−0.1030	0.1993	0.150*	0.50
C42	0.5000	0.0141 (4)	0.2500	0.0587 (14)	
C43	0.54010 (18)	0.0576 (4)	0.2095 (3)	0.0852 (15)	
H43	0.5679	0.0270	0.1809	0.102*	
C44	0.5404 (3)	0.1462 (5)	0.2097 (5)	0.152 (4)	
H44	0.5686	0.1767	0.1820	0.182*	
C45	0.5000	0.1894 (7)	0.2500	0.192 (8)	
H45	0.5000	0.2504	0.2500	0.230*	

### Atomic displacement parameters (Å^2^)

**Table:**

	*U*^11^	*U*^22^	*U*^33^	*U*^12^	*U*^13^	*U*^23^
Sn1	0.01857 (9)	0.01607 (9)	0.01429 (7)	0.00037 (6)	0.00206 (6)	−0.00086 (5)
Sn2	0.01640 (9)	0.01663 (9)	0.01515 (7)	0.00063 (6)	−0.00022 (5)	0.00075 (5)
Sn3	0.01802 (9)	0.01440 (8)	0.01468 (7)	0.00077 (6)	0.00034 (5)	0.00074 (5)
O1	0.0203 (8)	0.0183 (8)	0.0139 (7)	0.0016 (7)	0.0006 (6)	0.0007 (6)
O2	0.0191 (9)	0.0152 (8)	0.0174 (7)	−0.0001 (6)	0.0011 (6)	0.0006 (6)
O3	0.0183 (8)	0.0176 (8)	0.0153 (7)	0.0017 (7)	0.0011 (6)	−0.0001 (6)
O4	0.0257 (10)	0.0258 (9)	0.0170 (8)	−0.0023 (7)	0.0014 (6)	−0.0034 (6)
O5	0.0232 (9)	0.0251 (9)	0.0182 (8)	−0.0016 (7)	−0.0017 (6)	−0.0006 (6)
C4	0.0278 (15)	0.0144 (12)	0.0197 (11)	0.0004 (10)	−0.0025 (9)	0.0003 (8)
C5	0.0328 (15)	0.0413 (16)	0.0180 (11)	−0.0001 (13)	−0.0033 (10)	−0.0061 (10)
O6	0.0219 (9)	0.0190 (9)	0.0224 (8)	−0.0017 (7)	−0.0022 (6)	0.0021 (6)
O7	0.0201 (9)	0.0190 (9)	0.0215 (8)	−0.0008 (7)	−0.0026 (6)	0.0011 (6)
C6	0.0226 (13)	0.0199 (13)	0.0160 (10)	−0.0010 (10)	0.0050 (9)	−0.0032 (8)
C7	0.0275 (14)	0.0239 (14)	0.0308 (13)	−0.0069 (11)	−0.0017 (10)	0.0029 (10)
O8	0.0306 (9)	0.0197 (9)	0.0168 (7)	−0.0008 (7)	0.0029 (6)	0.0020 (6)
O9	0.0318 (10)	0.0205 (9)	0.0170 (8)	−0.0015 (7)	0.0063 (6)	0.0001 (6)
C8	0.0182 (12)	0.0245 (14)	0.0178 (11)	−0.0024 (10)	0.0013 (8)	0.0020 (9)
C9	0.0407 (16)	0.0284 (14)	0.0227 (12)	−0.0011 (12)	0.0077 (10)	0.0058 (10)
C11	0.0246 (14)	0.0245 (14)	0.0358 (14)	0.0031 (11)	0.0037 (10)	−0.0072 (10)
C12	0.0314 (15)	0.0227 (13)	0.0275 (12)	0.0040 (11)	−0.0004 (10)	−0.0052 (10)
C13	0.0445 (19)	0.0307 (17)	0.0562 (19)	0.0132 (14)	−0.0109 (14)	−0.0158 (13)
C14	0.079 (3)	0.0328 (18)	0.054 (2)	0.0212 (17)	−0.0220 (18)	−0.0145 (14)
C21	0.0253 (14)	0.0320 (15)	0.0292 (13)	0.0101 (11)	0.0033 (10)	0.0035 (10)
C22	0.0278 (17)	0.0427 (19)	0.086 (2)	0.0041 (14)	0.0221 (16)	−0.0081 (17)
C23	0.0265 (18)	0.061 (2)	0.103 (3)	0.0036 (16)	0.0147 (18)	−0.023 (2)
C24	0.043 (2)	0.077 (3)	0.155 (5)	0.006 (2)	0.027 (3)	−0.050 (3)
C31	0.0211 (13)	0.0236 (13)	0.0239 (11)	0.0047 (10)	0.0001 (9)	0.0029 (9)
C32	0.0305 (16)	0.0216 (15)	0.0356 (17)	0.0049 (12)	0.0078 (14)	0.0064 (12)
C33	0.038 (2)	0.0326 (18)	0.055 (2)	0.0124 (16)	0.0152 (18)	0.0107 (18)
C34	0.076 (3)	0.033 (2)	0.059 (2)	0.0217 (19)	0.030 (2)	0.0140 (16)
C35	0.0211 (13)	0.0236 (13)	0.0239 (11)	0.0047 (10)	0.0001 (9)	0.0029 (9)
C36	0.0305 (16)	0.0216 (15)	0.0356 (17)	0.0049 (12)	0.0078 (14)	0.0064 (12)
C37	0.038 (2)	0.0326 (18)	0.055 (2)	0.0124 (16)	0.0152 (18)	0.0107 (18)
C38	0.076 (3)	0.033 (2)	0.059 (2)	0.0217 (19)	0.030 (2)	0.0140 (16)
C41	0.057 (4)	0.095 (6)	0.143 (7)	0.000	−0.031 (4)	0.000
C42	0.041 (3)	0.085 (4)	0.046 (3)	0.000	−0.018 (2)	0.000
C43	0.050 (3)	0.138 (5)	0.064 (3)	−0.024 (3)	−0.0227 (19)	0.014 (3)
C44	0.103 (6)	0.153 (8)	0.184 (7)	−0.064 (5)	−0.090 (5)	0.086 (6)
C45	0.141 (12)	0.086 (8)	0.32 (2)	0.000	−0.161 (13)	0.000

### Geometric parameters (Å, º)

**Table:**

Sn1—O1	2.0969 (15)	C14—H14A	0.9800
Sn1—O2	2.0800 (15)	C14—H14B	0.9800
Sn1—O3	2.0957 (15)	C14—H14C	0.9800
Sn1—O4	2.1614 (15)	C21—C22	1.523 (4)
Sn1—O9	2.1665 (15)	C21—H21A	0.9900
Sn1—C11	2.129 (2)	C21—H21B	0.9900
Sn2—O1	2.0903 (15)	C22—C23	1.527 (4)
Sn2—O2	2.0843 (15)	C22—H22A	0.9900
Sn2—O3^i^	2.0882 (14)	C22—H22B	0.9900
Sn2—O5	2.1606 (15)	C23—C24	1.494 (5)
Sn2—O6	2.1715 (15)	C23—H23A	0.9900
Sn2—C21	2.129 (2)	C23—H23B	0.9900
Sn3—O1^i^	2.0845 (14)	C24—H24A	0.9800
Sn3—O2	2.0909 (15)	C24—H24B	0.9800
Sn3—O3	2.0885 (15)	C24—H24C	0.9800
Sn3—O7^i^	2.1724 (15)	C31—C32	1.516 (2)
Sn3—O8	2.1697 (14)	C31—H31A	0.9900
Sn3—C31	2.125 (2)	C31—H31B	0.9900
O1—Sn3^i^	2.0845 (14)	C32—C33	1.516 (2)
O3—Sn2^i^	2.0882 (14)	C32—H32A	0.9900
O4—C4	1.260 (3)	C32—H32B	0.9900
O5—C4	1.267 (3)	C33—C34	1.516 (2)
C4—C5	1.501 (3)	C33—H33A	0.9900
C5—H5A	0.9800	C33—H33B	0.9900
C5—H5B	0.9800	C34—H34A	0.9800
C5—H5C	0.9800	C34—H34B	0.9800
O6—C6	1.259 (3)	C34—H34C	0.9800
O7—C6	1.266 (3)	C36—C37	1.516 (2)
O7—Sn3^i^	2.1724 (15)	C36—H36A	0.9900
C6—C7	1.501 (3)	C36—H36B	0.9900
C7—H7A	0.9800	C37—C38	1.516 (2)
C7—H7B	0.9800	C37—H37A	0.9900
C7—H7C	0.9800	C37—H37B	0.9900
O8—C8	1.267 (3)	C38—H38A	0.9800
O9—C8	1.256 (3)	C38—H38B	0.9800
C8—C9	1.503 (3)	C38—H38C	0.9800
C9—H9A	0.9800	C41—C42	1.498 (8)
C9—H9B	0.9800	C41—H41A	0.9800
C9—H9C	0.9800	C41—H41B	0.9800
C11—C12	1.524 (3)	C41—H41C	0.9800
C11—H11A	0.9900	C42—C43^ii^	1.360 (5)
C11—H11B	0.9900	C42—C43	1.360 (5)
C12—C13	1.512 (4)	C43—C44	1.381 (9)
C12—H12A	0.9900	C43—H43	0.9500
C12—H12B	0.9900	C44—C45	1.362 (10)
C13—C14	1.525 (4)	C44—H44	0.9500
C13—H13A	0.9900	C45—C44^ii^	1.362 (10)
C13—H13B	0.9900	C45—H45	0.9500
			
O2—Sn1—O3	77.96 (6)	C13—C12—C11	113.6 (2)
O2—Sn1—O1	77.88 (6)	C13—C12—H12A	108.9
O3—Sn1—O1	104.78 (6)	C11—C12—H12A	108.9
O2—Sn1—C11	177.18 (8)	C13—C12—H12B	108.9
O3—Sn1—C11	100.30 (8)	C11—C12—H12B	108.9
O1—Sn1—C11	100.53 (8)	H12A—C12—H12B	107.7
O2—Sn1—O4	87.87 (6)	C12—C13—C14	113.0 (3)
O3—Sn1—O4	159.07 (6)	C12—C13—H13A	109.0
O1—Sn1—O4	86.89 (6)	C14—C13—H13A	109.0
C11—Sn1—O4	94.39 (8)	C12—C13—H13B	109.0
O2—Sn1—O9	87.75 (6)	C14—C13—H13B	109.0
O3—Sn1—O9	85.78 (6)	H13A—C13—H13B	107.8
O1—Sn1—O9	159.73 (6)	C13—C14—H14A	109.5
C11—Sn1—O9	94.36 (8)	C13—C14—H14B	109.5
O4—Sn1—O9	78.27 (6)	H14A—C14—H14B	109.5
O2—Sn2—O3^i^	104.24 (6)	C13—C14—H14C	109.5
O2—Sn2—O1	77.93 (6)	H14A—C14—H14C	109.5
O3^i^—Sn2—O1	78.22 (5)	H14B—C14—H14C	109.5
O2—Sn2—C21	99.96 (8)	C22—C21—Sn2	114.34 (19)
O3^i^—Sn2—C21	100.30 (8)	C22—C21—H21A	108.7
O1—Sn2—C21	176.96 (8)	Sn2—C21—H21A	108.7
O2—Sn2—O5	86.58 (6)	C22—C21—H21B	108.7
O3^i^—Sn2—O5	160.42 (6)	Sn2—C21—H21B	108.7
O1—Sn2—O5	88.41 (6)	H21A—C21—H21B	107.6
C21—Sn2—O5	93.67 (8)	C21—C22—C23	113.6 (3)
O2—Sn2—O6	160.09 (6)	C21—C22—H22A	108.9
O3^i^—Sn2—O6	86.44 (6)	C23—C22—H22A	108.9
O1—Sn2—O6	88.13 (6)	C21—C22—H22B	108.9
C21—Sn2—O6	94.44 (8)	C23—C22—H22B	108.9
O5—Sn2—O6	78.81 (6)	H22A—C22—H22B	107.7
O1^i^—Sn3—O3	78.34 (6)	C24—C23—C22	113.5 (3)
O1^i^—Sn3—O2	103.89 (6)	C24—C23—H23A	108.9
O3—Sn3—O2	77.88 (6)	C22—C23—H23A	108.9
O1^i^—Sn3—C31	102.40 (7)	C24—C23—H23B	108.9
O3—Sn3—C31	175.48 (7)	C22—C23—H23B	108.9
O2—Sn3—C31	97.63 (7)	H23A—C23—H23B	107.7
O1^i^—Sn3—O8	160.23 (6)	C23—C24—H24A	109.5
O3—Sn3—O8	88.49 (6)	C23—C24—H24B	109.5
O2—Sn3—O8	87.42 (6)	H24A—C24—H24B	109.5
C31—Sn3—O8	91.90 (7)	C23—C24—H24C	109.5
O1^i^—Sn3—O7^i^	86.96 (6)	H24A—C24—H24C	109.5
O3—Sn3—O7^i^	87.47 (6)	H24B—C24—H24C	109.5
O2—Sn3—O7^i^	159.40 (6)	C32—C31—Sn3	116.47 (17)
C31—Sn3—O7^i^	97.01 (7)	C32—C31—H31A	108.2
O8—Sn3—O7^i^	77.69 (6)	Sn3—C31—H31A	108.2
Sn3^i^—O1—Sn2	100.00 (6)	C32—C31—H31B	108.2
Sn3^i^—O1—Sn1	132.51 (7)	Sn3—C31—H31B	108.2
Sn2—O1—Sn1	99.79 (6)	H31A—C31—H31B	107.3
Sn1—O2—Sn2	100.55 (6)	C31—C32—C33	112.4 (3)
Sn1—O2—Sn3	100.39 (6)	C31—C32—H32A	109.1
Sn2—O2—Sn3	133.30 (7)	C33—C32—H32A	109.1
Sn2^i^—O3—Sn3	99.94 (6)	C31—C32—H32B	109.1
Sn2^i^—O3—Sn1	132.08 (7)	C33—C32—H32B	109.1
Sn3—O3—Sn1	99.95 (6)	H32A—C32—H32B	107.9
C4—O4—Sn1	129.87 (14)	C34—C33—C32	113.6 (3)
C4—O5—Sn2	129.71 (15)	C34—C33—H33A	108.9
O4—C4—O5	125.6 (2)	C32—C33—H33A	108.9
O4—C4—C5	117.3 (2)	C34—C33—H33B	108.9
O5—C4—C5	117.0 (2)	C32—C33—H33B	108.9
C4—C5—H5A	109.5	H33A—C33—H33B	107.7
C4—C5—H5B	109.5	C37—C36—H36A	107.8
H5A—C5—H5B	109.5	C37—C36—H36B	107.8
C4—C5—H5C	109.5	H36A—C36—H36B	107.1
H5A—C5—H5C	109.5	C36—C37—C38	116 (3)
H5B—C5—H5C	109.5	C36—C37—H37A	108.3
C6—O6—Sn2	129.60 (15)	C38—C37—H37A	108.3
C6—O7—Sn3^i^	129.45 (14)	C36—C37—H37B	108.3
O6—C6—O7	126.0 (2)	C38—C37—H37B	108.3
O6—C6—C7	116.8 (2)	H37A—C37—H37B	107.4
O7—C6—C7	117.2 (2)	C37—C38—H38A	109.5
C6—C7—H7A	109.5	C37—C38—H38B	109.5
C6—C7—H7B	109.5	H38A—C38—H38B	109.5
H7A—C7—H7B	109.5	C37—C38—H38C	109.5
C6—C7—H7C	109.5	H38A—C38—H38C	109.5
H7A—C7—H7C	109.5	H38B—C38—H38C	109.5
H7B—C7—H7C	109.5	C42—C41—H41A	109.5
C8—O8—Sn3	128.85 (14)	C42—C41—H41B	109.5
C8—O9—Sn1	131.40 (14)	H41A—C41—H41B	109.5
O9—C8—O8	125.3 (2)	C42—C41—H41C	109.5
O9—C8—C9	117.4 (2)	H41A—C41—H41C	109.5
O8—C8—C9	117.3 (2)	H41B—C41—H41C	109.5
C8—C9—H9A	109.5	C43^ii^—C42—C43	120.3 (7)
C8—C9—H9B	109.5	C43^ii^—C42—C41	119.9 (3)
H9A—C9—H9B	109.5	C43—C42—C41	119.9 (3)
C8—C9—H9C	109.5	C42—C43—C44	120.0 (6)
H9A—C9—H9C	109.5	C42—C43—H43	120.0
H9B—C9—H9C	109.5	C44—C43—H43	120.0
C12—C11—Sn1	115.28 (17)	C45—C44—C43	119.5 (9)
C12—C11—H11A	108.5	C45—C44—H44	120.2
Sn1—C11—H11A	108.5	C43—C44—H44	120.2
C12—C11—H11B	108.5	C44^ii^—C45—C44	120.7 (12)
Sn1—C11—H11B	108.5	C44^ii^—C45—H45	119.7
H11A—C11—H11B	107.5	C44—C45—H45	119.7
			
Sn2—O2—Sn3—O1^i^	−25.88 (11)	O1^i^—Sn1^i^—O3^i^—Sn2	24.53 (11)
O2—Sn3—O1^i^—Sn1^i^	24.87 (11)	Sn1^i^—O3^i^—Sn2—O2	−24.89 (11)
Sn3—O1^i^—Sn1^i^—O3^i^	−24.64 (11)	O3^i^—Sn2—O2—Sn3	25.96 (11)

Symmetry codes: (i) −*x*+1/2, −*y*+3/2, −*z*; (ii) −*x*+1, *y*, −*z*+1/2.
